# The impact of florfenicol treatment on the microbial populations present in the gill, intestine, and skin of channel catfish (*Ictalurus punctatus*)

**DOI:** 10.1186/s42523-025-00433-9

**Published:** 2025-06-20

**Authors:** Hongye Wang, Lina Sheng, Zeinab Yazdi, Xiran Li, Zhuosheng Liu, Sushumna Canakapalli, Yi Zhou, Chao Liao, Shiva Emami, Anita M. Kelly, Luke A. Roy, Esteban Soto, Luxin Wang

**Affiliations:** 1 Department of Food Science and Technology, University of California Davis, One Shields Avenue, Davis, CA 95616-5270 USA; 2 School of Food Science and Technology, International Joint Laboratory on Food Safety, Synergetic Innovation Center of Food Safety and Quality Control, Jiangnan University, Wuxi, 214122 Jiangsu People’s Republic of China; 3 School of Veterinary Medicine, University of California Davis, Davis, CA 95616 USA; 4 School of Fisheries, Aquaculture & Aquatic Sciences, Auburn University, Auburn, AL 3684 USA

**Keywords:** Antibiotic-resistant bacteria, Florfenicol, Fish, Catfish microbiome

## Abstract

**Background:**

Florfenicol is a broad-spectrum antimicrobial approved in many countries for treating bacterial infections in production animals. Although florfenicol has been widely used in the US catfish industry, its impact on the native microbiota within catfish tissues remains largely unknown. Florfenicol treatment is followed by a mandatory withdrawal period to ensure drug residues fall below regulatory limits before harvest. This interval also allows for the potential recovery of the native microbiota. In particular, the skin and gill microbiota have often been overlooked in aquaculture microbiome research. Moreover, the dynamics of microbial communities and resistome profiles following drug withdrawal are still poorly understood, despite their ecological significance.

**Results:**

A significant increase in intestinal microbial diversity was observed at the end of the withdrawal period. The highest alpha diversity (Shannon index) was observed in catfish intestines. This increase indicated the restoration of the normal microbiota in catfish intestine. The predominant bacterial phyla shared among catfish gill, intestine, and skin are Proteobacteria (62%), Bacteroidetes (18%), Actinobacteriota (12%), Firmicutes (3%), Patescibacteria (2%), and Verrucomicrobiota (1%). Florfenicol application can have lasting effects through the withdrawal period, particularly altering the intestinal microbial community.

**Conclusion:**

The result of this study underscores the impact of florfenicol treatment on the bacterial landscape and antibiotic resistance in catfish, highlighting significant changes in microbial composition in the catfish intestine and at the end of the withdrawal period. These findings address the need for monitoring and managing antibiotic resistance in fish farming environments.

**Supplementary Information:**

The online version contains supplementary material available at 10.1186/s42523-025-00433-9.

## Background

According to the Food and Agriculture Organization (FAO) of the United Nations, the global demand for meat will reach 455 M metric tons by 2050 [1]. As “the world’s most efficient protein generator,” aquaculture is growing faster than any other major food sector and is forecasted to reach 140 M metric tons by 2050 [2]. Even if more than one-third of protein production comes from aquaculture by 2050, aquaculture still requires less feed crop and land than livestock [3]. Bacterial diseases are the leading cause of economic losses in the U.S. catfish industry. Taking Alabama as an example, disease-related losses total approximately $11.1 million annually, about 9.5% of food-size catfish sales, with bacterial infections alone responsible for over 83% of these losses. This indicates the substantial impact of bacterial pathogens on catfish health and aquaculture production [4, 5]. To treat bacterial infections and reduce economic losses, the antimicrobial-medicated feed has been used for treating both warm-water fish diseases and cold-water fish infections including catfish [6].

In the US, three antibiotic-medicated feeds are approved for aquaculture in certain species, including florfenicol, oxytetracycline dihydrate, and sulfadimethoxine/ormetoprim [7]. In catfish farming, enteric septicemia of catfish (ESC) and columnaris are the top two economically important diseases; 78.1% of all operations and 42.1% of all ponds experienced problems with ESC/columnaris, and each causes fish loss of more than 15% of all operations [8]. ESC caused by *Edwardsiella ictaluri* is the most prevalent disease. It occurs when water temperatures fluctuate between 22 and 28 °C during the late spring and early fall, which overlaps with the catfish growing season from April to October at 25–30 °C [6]. Infected fish present higher mortalities at warmer temperatures [9]. In the US, florfenicol (Aquaflor®) and sulfadimethoxine/ormetoprim (Romet®) are approved for use in medicated feed to treat ESC, and sulfadimethoxine/ormetoprim is also used to treat columnaris outbreaks [7]. In 2024, the U.S. Food and Drug Administration approved Paqflor (florfenicol), the first generic florfenicol drug for controlling mortality in certain species of freshwater-reared fish, providing an additional option for disease management in aquaculture [10]. Florfenicol is more commonly used than sulfadimethoxine/ormetoprim as it can also treat columnaris disease in other fish species, so more feed distributors carry the product [6]. The recommended dosage of florfenicol to control mortality in catfish due to ESC is 10 mg per kg of body weight for 10 consecutive days [11]. A withdrawal period of 12 days [11] is required prior to slaughter, and the tolerance for florfenicol amine in the muscle or muscle/skin is 1 ppm.

Regardless of the host, antimicrobial usage exerts a significant selective pressure, leading to alterations of microbial populations and providing a selective advantage to antimicrobial resistant bacteria [12]. Many antimicrobial agents used in human medicine are also administered to food animals for treatment. This overlap usage increases the risk of antimicrobial resistant bacteria [13, 14]. Previous studies have explored the impact of medicated feed on aquatic organisms and their rearing environments at the end of treatment, revealing a decrease in intestinal microbiota richness and diversity among catfish [15, 16]. This was indicated by shifts in microbial composition, notably an increased prevalence of the phylum Proteobacteria, and a reduction in Firmicutes and Bacteroidetes [15, 16]. Florfenicol-medicated feed also enriched the relative abundance of opportunistic pathogens including *Plesiomonas* and *Aeromonas* species in catfish [16]. However, the impact of the withdrawal period post-antibiotic treatment remains markedly understudied. Once the animal stops receiving the antibiotic, the drug elimination during the withdrawal period may trigger an upsurge in multi-drug resistance among certain pathogenic bacteria, potentially leading to a belated resistome response or facilitating horizontal gene transfer [17]. This underscores the need for comprehensive investigations into microbial dynamics throughout the drug withdrawal period. The effect of florfenicol on different parts of the catfish (e.g., skin, gill) during the withdrawal period, along with the impact of water temperature on the development of AMR, presents a significant research gap, warranting further exploration.

Previous studies showed that florfenicol-medicated feed altered the resistome of water in catfish tanks; both intra- and inter-population horizontal transfer of the antibiotic resistance gene was observed [18]. Other studies have evaluated the impact of florfenicol-medicated feed on catfish gut microbiota [15, 16], but the effect of florfenicol on the skin and gill microbiome and the development of AMR is scarce. The treatment efficacy of florfenicol has been proven at a wide range of water temperatures; on the other hand, it is well known that water temperature can influence the fate of drugs in water. In this case, the impact of this drug on microbial composition and AMR formation at different water temperatures at which the drugs are applied remains unclear. While it is known that florfenicol can alter the microbial composition in both catfish and their rearing water after treatment, the dynamic in microbial and resistome profiles after drug withdrawal remains poorly understood despite their ecological importance. This study will contribute to understanding the comprehensive patterns and dynamics of the microbiome and resistome of catfish in response to florfenicol treatment at varying temperatures, from catfish after florfenicol treatment to catfish after the withdrawal period.

## Materials and methods

### Catfish preparation and experimental tank setup

Twenty-four 132.5 L tanks were set up at the Center for Aquatic Biology and Aquaculture (CABA) of the University of California, Davis (Davis, California). The following animal handling and treatment protocols were approved by the Institutional Animal Care and Use Committee (protocol number #21,745). Four replicate tanks were used for each treatment regime. Individual tanks were filled with well water and equipped with airlift biofilters. Since catfish in different treatments and replicates were fed different medicated feeds, individual tank biofilters were the most practical option for maintaining catfish for this experiment. Briefly, each tank airlift biofilter was used according to Roy et al. [19] and constructed of 5 cm diameter PVC pipe, a 9.5 mm inside diameter airline, and a 15 cm ceramic air diffuser supplied with aeration from a 1/3 HP Sweetwater regenerative blower (Aquatic Ecosystems, Inc., Lake Apopka, Florida, USA). Fish spawning mat material was wrapped around the PVC pipe to provide a substrate for bacterial growth (Supplemental Fig. S1A). Underneath the spawning substrate, there were several slits in the PVC pipe to allow entry of water into the bottom of the airlift biofilter to provide water flow through and up the airlift biofilter. Three weeks (21 days) before the experiment, all tanks were seeded with ammonium chloride (Fritz PRO, Mesquite, TX) following the manufacturer’s instructions to initiate the growth of nitrifying bacteria on the airlift biofilter.

To evaluate the impact of florfenicol on microbial communities, channel catfish (*Ictalurus punctatus*) fingerlings (average weight ca. 7.35 g/fish, median 6.82 g/fish; ~ 9 cm long) were stocked at a density of 25 fish per tank. Catfish were acclimatized for 14 days. Before applying florfenicol-medicated feed, catfish were fed a commercial feed at a typical production rate (ca. 2.5% of body weight per day) for two weeks to establish and stabilize the microbial communities in the production system. The fish were fed once per day; uneaten feed was removed from the tanks 30 min after feeding to avoid spoilage and water quality deterioration. This experiment was conducted at three temperatures (20, 25, and 30 °C) using aquarium heaters (Amazon, Bellevue, WA). Florfenicol (Aquaflor, Merck, Lawrence, KS) medicated feed prepared by a commercial feed mill was given at 10 mg/kg body weight and fed at a 2% body weight per day for a period of 10 days, followed by a 12-day withdrawal period. Dissolved oxygen was maintained at ca. 6 mg/L for all temperatures during the experimental period. Water quality was monitored using an API Freshwater master test kit (Chalfont, PA) every 5 to 7 days, including each sampling point (before florfenicol treatment, after florfenicol treatment, and after the withdrawal period). The water quality parameters (pH, unionized ammonia in mg/L, and nitrite in mg/L) are summarized in Supplementary Tables S1, S2, and S3. When unionized ammonia were outside the range of 0.1 mg/L [20], water changes of 5 L were made to prevent water quality deterioration.

### Drug residue analysis of water samples

To determine the drug residue in water, water samples (100 mL each) were collected from each tank maintained at 20, 25, or 30 °C on the day before florfenicol treatment (day 0), after 10 days of florfenicol treatment (day 10), and after the 12-day withdrawal period (day 24). The extraction of florfenicol from water samples was carried out using solid phase extraction (SPE). Briefly, 25 mL of each water sample was added into 40 mL clear glass vials (VWR, PA) using glass pipettes to prepare the water samples. Samples were then spiked with 40 μL of a surrogate mix (500 ng/mL; TRIM-D3, CTC, CAP-D5, SMX-D4). MiliQ water spiked with the surrogate mix was used as the control alongside the testing samples. To carry out the analysis, 1340 µL of 0.05 M EDTA were added to each sample, and aliquots were allowed to equilibrate for 1 h with occasional shaking. Samples’ pH was adjusted to pH = 2.5 by adding 15 µL of HCl (37%). OASIS HLB cartridges (Waters, 60 mg, 3 mL) were conditioned using 6 mL of methanol, 6 mL of reagent water, and 6 mL of pH = 2.5 water. Water samples were then loaded into a conditioned HLB cartridge. The columns were washed with water (6 mL) and dried under the vacuum for 5 min. Antibiotics were then eluted using methanol (6 mL). The eluents were dried under the nitrogen and reconstituted in 1 mL of methanol. Samples were vortexed for 3 min, transferred into 2 mL tubes, and centrifuged at 13,523 × g (12,000 rpm, Eppendorf, 5424 R) for 2 min. Samples were then filtered through centrifuge tubes containing 0.1 µm filters (Ultrafree-MC-VV; PVDF 0.1 µm; Millipore Sigma, MA) by centrifugation (10 min at 13,523 × g, 12,000 rpm). The extracts were transferred into an insert containing LC vials and stored at − 80 °C for LC–MS/MS analysis.

### LC–MS/MS analysis

Antibiotic analysis was performed using Agilent ultra-high pressure liquid chromatography coupled with a 6460 Agilent triple quad (UPLC-MS/MS) (Agilent Technologies Inc., CA). Chromatographic separation of the antibiotic mixture was performed with the AQUITY BEH C18 column (100 × 2.1 mm, 1.8 µm, Waters Corp, MA). The column temperature was set at 30 °C, and the flow rate was 0.3 mL/min. Formic acid (0.1% in water) and formic acid (0.1% in acetonitrile) were used as mobile phases A and B, respectively. MS/MS analysis was performed using the Agilent Jetstream electrospray ionization (ESI). MS source parameters were set as follows: sheath gas temperature of 375 °C, sheath gas flow of 11 L/min, drying gas temperature of 250 °C, nozzle voltage of 0 V, nebulizer gas pressure of 40 psi, and capillary voltage of 3500 V. Collision induced dissociation was carried out by using nitrogen at the collision cell. Specific MS/MS parameters are shown in Supplemental Table S4. Concentrations of florfenicol were determined by the internal standard (CAP-D5) calibration and the external calibration curve made by standards containing florfenicol dissolved in methanol (in a range of 0.5–800 ng/mL). A quadratic regression model and 1/x weighting factor were used to create the standard curve.

### Catfish sample collection

**Fig. 1:**
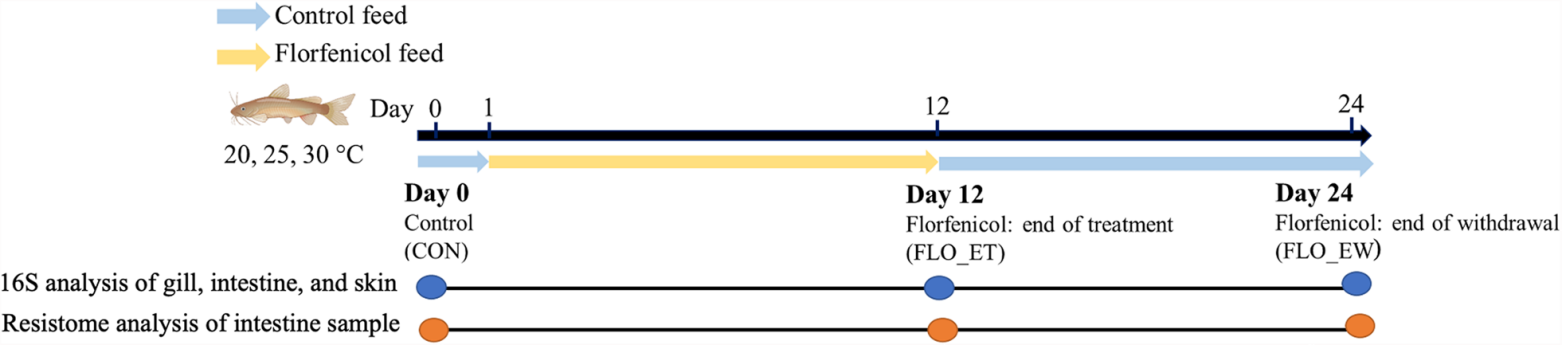
Experimental design of this study

At each sampling point, three catfish were taken from each tank before treatment (day 0; control), at the end of treatment (day 12), and at the end of withdrawal (day 24) (Fig. 1) for DNA extraction. Catfish taken from tanks were euthanized in tricaine methanesulfonate solution (MS 222, 500 mg/L, TCI America, Portland, OR) buffered with sodium bicarbonate (Arm & Hammer, Trenton, NJ). Microorganisms present on fish were collected by swabbing the gill arches, intestine content, and fish skin with FLOQSwabs® (Copan Diagnostics, Murrieta, CA). Due to the small size of the catfish fingerlings, the entire intestinal content was collected without separating specific gut regions. Swabs used for DNA extraction were placed in the PowerBead Pro tubes provided in the DNeasy® PowerSoil® Pro kit individually (Qiagen, Valencia, CA) for storage (− 20 °C) and DNA extraction.

### Microbial profile analysis by 16S rRNA gene sequencing

Swab samples from catfish gill, intestine and skin were subjected to DNA extraction by using the DNeasy® PowerSoil® Pro kit according to the manufacturer’s instructions. The purity and concentration of DNA were evaluated with a plate reader spectrophotometer (NanoDrop Technologies, DE, USA) at 260 and 280 nm. Further quantification of DNA was performed by using a Qubit fluorometer (Thermo Fisher, MA).

In total, 108 high-quality DNA samples were collected from the catfish gill, intestine and skin in control and florfenicol treatment groups at 20, 25, and 30 °C (Supplemental Table S5). The bacterial 16S rRNA gene-targeted sequencing library waxfs prepared using the QIAseq 16S Panel and Index kits (Qiagen). The bacterial 16S primers were used to amplify the V3-V4 region of the 16S rRNA gene. The final pooled library was evaluated by the Agilent Bioanalyzer 2100 System with Agilent DNA 1000 kits (Santa Clara, CA) and quantified with TapeStation (Agilent) and Qubit. The final library was sequenced on Illumina MiSeq with a Version 3 reagent kit (600 cycles). The sequencing was performed with > 10% PhiX spike-in. For analysis, raw fastq files were de-multiplexed, quality-filtered using QIIME2 [21]. Unique amplicon sequences were inferred from raw reads, and chimeric sequences were removed using the DADA2 pipeline [22]. Chimera-free sequences were processed for downstream analysis. Taxonomy annotation was performed using Uclust against the SILVA 16S rDNA database V138.

### Antibiotic resistance gene analysis by shotgun metagenomics sequencing

To understand changes in the antibiotic resistance gene (ARG) profile caused by the florfenicol treatment and treatment temperatures, samples collected from catfish intestines (n = 18; Supplemental Table S5) with high DNA quality were submitted for shotgun metagenomic sequencing. The library was prepared and sequenced by the UC Davis Genome Center. Briefly, barcode-indexed sequencing libraries were generated from genomic DNA samples sheared with an E220 Focused Ultrasonicator (Covaris, Woburn, MA). Sheared DNA was converted to sequencing libraries using the KAPA Hyper Prep Kit (Kapa Biosystems-Roche, Basel, Switzerland). The libraries were amplified with 5 PCR cycles and analyzed with a Bioanalyzer 2100 instrument (Agilent, Santa Clara, CA). The constructed library from each sample was quantified by fluorometry on Qubit (LifeTechnologies, Carlsbad, CA), and combined into pools at equimolar ratios. The pools were quantified by qPCR with the Kapa Library Quant kit (Kapa Biosystems-Roche) and each pool was sequenced on an Illumina NovaSeq (Illumina, San Diego, CA) with paired-end 150 bp reads.

For data analysis, raw sequencing reads were checked with FastQC (version 0.11.8) followed by quality-trimming with Trimmomatic (version 0.39) [23] with default parameters. After that, Bowtie 2 (version 2.4.2) [24] was used to remove the host information from the trimmed reads by aligning reads to the catfish host genome. The filtered and host information-removed reads were aligned to the MEGARes (version 3.0) to profile resistome structures following the AMR + + pipeline [https://www.meglab.org] [25] with modification. Briefly, the reads were mapped to the database (MEGARes) by using BWA-MEM (version 2.0) [26] with the default setting. The resulting SAM file was analyzed using the ResistomeAnalyzer with an 80% gene fraction threshold for ARG characterization. The data at the gene level from AMR + + was used to calculate the ARG richness and visualization by waffle plot, and the class level was used for visualization on heatmaps.

### Statistical analyses

For the 16S rRNA gene sequencing data analysis, within-community diversity (alpha diversity) was calculated using observed features and the Shannon index of species, followed by the Kruskal–Wallis test. Beta-diversity was measured with ecological phylogenetic Unifrac distances [27]. Principle-coordinate analysis (PCoA) was performed to determine whether samples associated with the same groups (catfish tissues, sampling times, and temperatures) clustered close to one another in multivariate space. Permutational multivariate analysis of variance (PERMANOVA) was used to test the statistical significance of group separation in PCoA with Benjamini–Hochberg FDR (BH-FDR). The Similarity percentage test (SIMPER) was used to find key microbial members contributing to the variation in community composition due to the florfenicol treatment*.* These analyses were run and visualization in R with packages including vegan (V2.6–4) and phyloseq (V1.42.0).

## Results

### Florfenicol levels in water and catfish weight

No florfenicol was detected in water samples collected from the control tanks. At the end of treatment, the concentration of florfenicol in water at 20, 25, and 30 °C was 15.17 ± 10.91, 16.68 ± 13.44. and 12.66 ± 6.24 ng/mL, respectively. Temperature did not significantly affect the florfenicol residue levels in water (*P* = 0.9590). The florfenicol residue level in the water decreased significantly (*P* = 0.009) after 12 days of withdrawal. At the end of withdrawal, the concentration of florfenicol in water at 20, 25, and 30 °C was 3.87 ± 6.31, 0.98 ± 0.40 and 3.64 ± 4.94 ng/mL, respectively, with no statistical difference observed among the three temperatures. The catfish weight was measured at different sampling points; the median weight ranged from 4.7 to 7.9 g (Supplemental Table S6). The survival proportion of catfish in control and florfenicol treatment groups is shown in the Supplemental Fig. S1B.

### Impacts of florfenicol treatment on microbial compositions associated with catfish

**Fig. 2:**
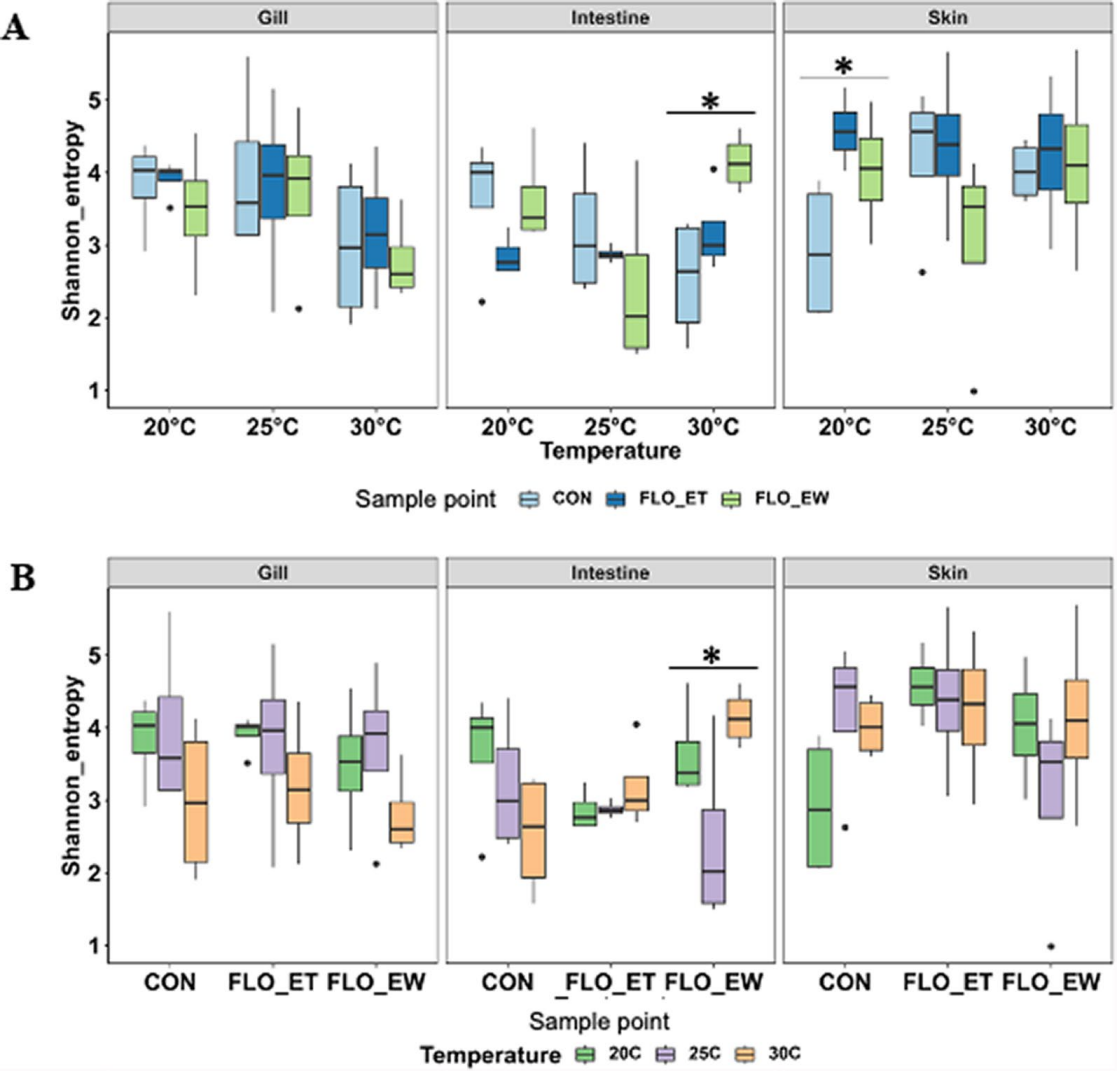
Boxplots depicting the Shannon index changes in catfish samples (gill, intestine, and skin) at different temperatures (**A**) and sampling points (**B**). Effects of florfenicol treatment and treatment temperatures were tested using Kruskal–Wallis analysis, and significant levels with Benjamini–Hochberg FDR adjusted *p-*value are indicated with *, which indicates significant differences with *p*-value < 0.05. CON: control; FLO_ET: florfenicol-end of treatment; FLO_EW: florfenicol-end of withdrawal

Downstream analysis was conducted using an average of 77,545 ± 49,578 chimera-free sequences for DNA samples collected from catfish gill, intestine, and skin. As shown in Fig. 2, the impact of florfenicol on the microbial populations (Shannon diversity) associated with catfish is sampling site- (gill, intestine, and skin), treatment temperature-, and sampling point dependent. When florfenicol was applied at 20 and 30 °C, significant impacts on the intestine and skin microbiota were observed (Fig. 2A). However, such an effect was not seen when florfenicol was applied at 25 °C. At 30 °C, the Shannon diversity of catfish intestine samples increased after the florfenicol treatment and continued increasing until the end of withdrawal (*P_*adjust < 0.05). For catfish skin samples, the highest Shannon index value was observed at the end of treatment at 20 °C (Fig. 2A). When looking into each sampling point (control, end of treatment, or end of withdrawal), treatment temperatures significantly (*P_*adjust < 0.05) impacted the Shannon index of microbiota present in catfish intestine samples at the end of withdrawal, with the highest value seen at 30 °C (Fig. 2B).

**Fig. 3:**
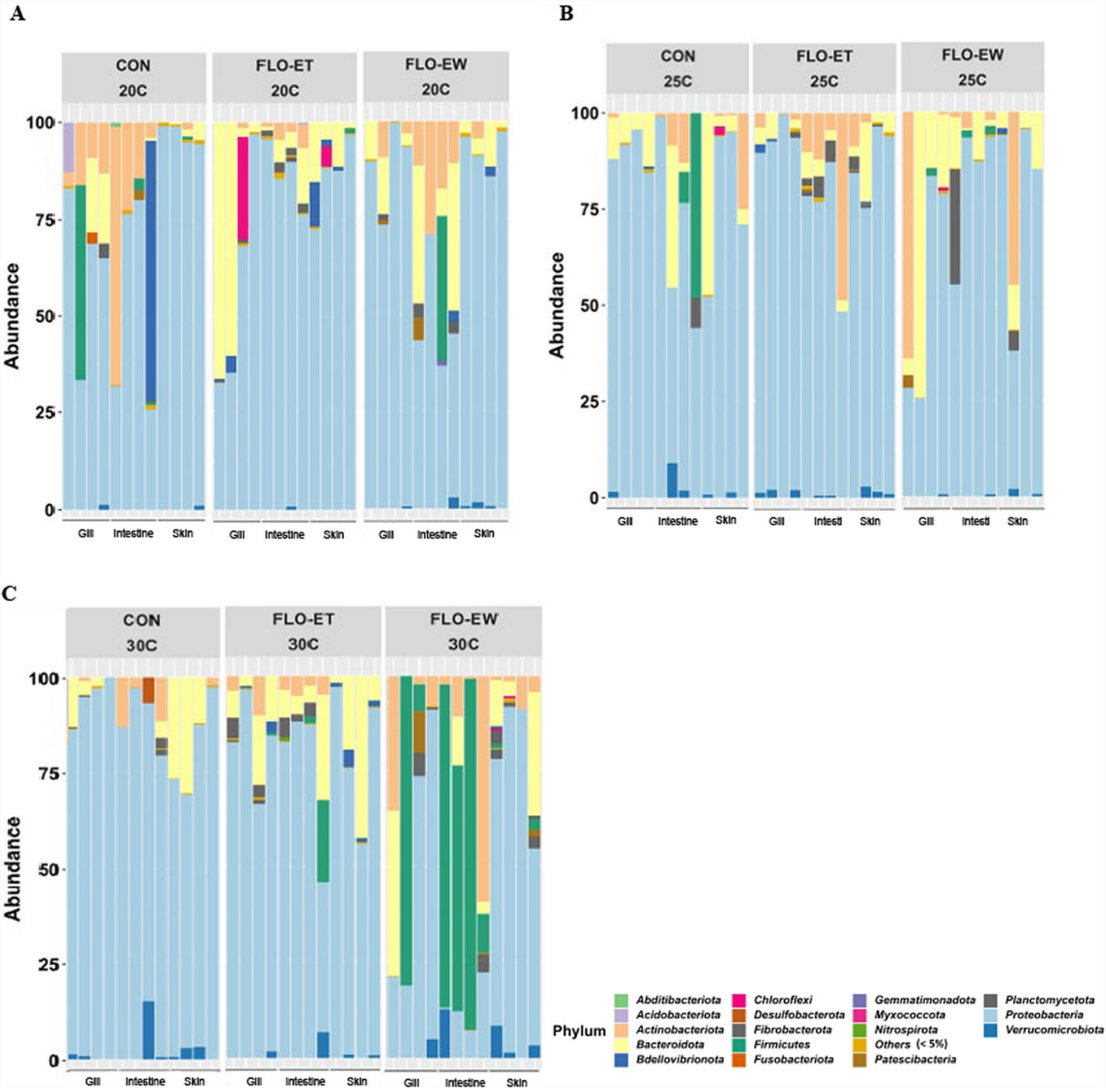
Bar plot depicting the relative abundance of microbial compositions, at the phylum level, of catfish gill, intestine, skin samples. Bacterial phyla which had a relative abundance of less than 0.5% were grouped into “Others”. Samples were separated by treatment scheme. CON: control group; FLO-ET: florfenicol-end of treatment; FLO-EW: florfenicol-end of withdrawal

Proteobacteria (62%), Bacteroidota (18%), Actinobacteriota (12%), Firmicutes (3%), Patescibacteria (2%), and Verrucomicrobiota (1%) were the major bacterial phyla observed in catfish gill, intestine, and skin (Fig. 3). These phyla represented over 98% of the relative abundances in all catfish samples. Proteobacteria had significantly greater relative abundances than other phyla regardless of the florfenicol treatment, rearing water temperature, and sample tissues (Fig. 3). The abundance of Firmicutes enriched in intestine samples at the end of withdrawal at 30 °C (Fig. 3).

**Fig. 4:**
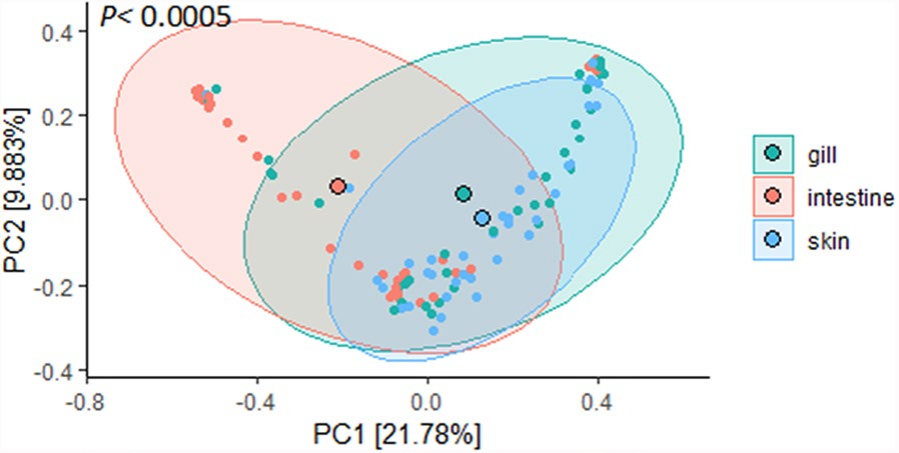
Weighted UniFrac distance-based principal-coordinate analysis (PCoA) of samples collected from the control and treatment groups at 20, 25, and 30 °C, including catfish gill, intestine, and skin. The centroid of each ellipse represents the group mean, and the shaded polygons, indicating the 95% confidence region of each cluster, are applied to differentiate sample types

Exploratory PCoA was used to analyze and compare the beta diversity of all samples (Fig. 4). The weighted UniFrac distance was used to calculate the relationship matrix associated with catfish's gill, intestine, and skin (PERMANOVA tested by adonis2; *p* < 0.0005). Ordination analysis revealed that 21.78% and 9.88% variances in bacterial composition were represented by the PC1 and PC2, respectively, with the microbial communities clustering distinctly between catfish gill, intestine, and skin. It can also be seen that the microbial populations associated with gill and skin were more overlapped based on the observation of the distance between the centroid of these two groups (Fig. 4), while the intestine samples had a more distinctive microbial structure.

Therefore, the beta diversity of microbial compositions in the catfish gill, intestine, and skin samples by florfenicol treatment and temperature was analyzed separately. In gill samples, florfenicol treatment significantly affected beta diversity at 25 °C but not at 20 or 30 °C (PERMANOVA; *P* = 0.0005; Supplemental Figure S2A). While temperatures significantly affected the beta diversity of gill samples in the control group (*P* = 0.0025; Supplemental Figure S2B), they did not significantly affect florfenicol-treated groups. For intestine samples, florfenicol treatment significantly altered the microbial structures at 20 and 30 °C (*P* = 0.0120; Supplemental Figure S2C). Within each sampling point (control, at the end of treatment, at the end of withdrawal), the significant impact of temperature was only seen at the end of withdrawal (*P* = 0.0160; Supplemental Figure S2D). In skin samples, florfenicol significantly influenced the skin microbial structures at 20 (*P* = 0.0165) and 30 °C (*P* = 0.0340; Supplemental Figure S2E). Within each sampling point, temperature altered the microbiota of skin samples collected before (*P* = 0.0005) and at the end of treatments (*P* = 0.0005) but not at the end of withdrawal (Supplemental FigureS2F).

**Fig. 5:**
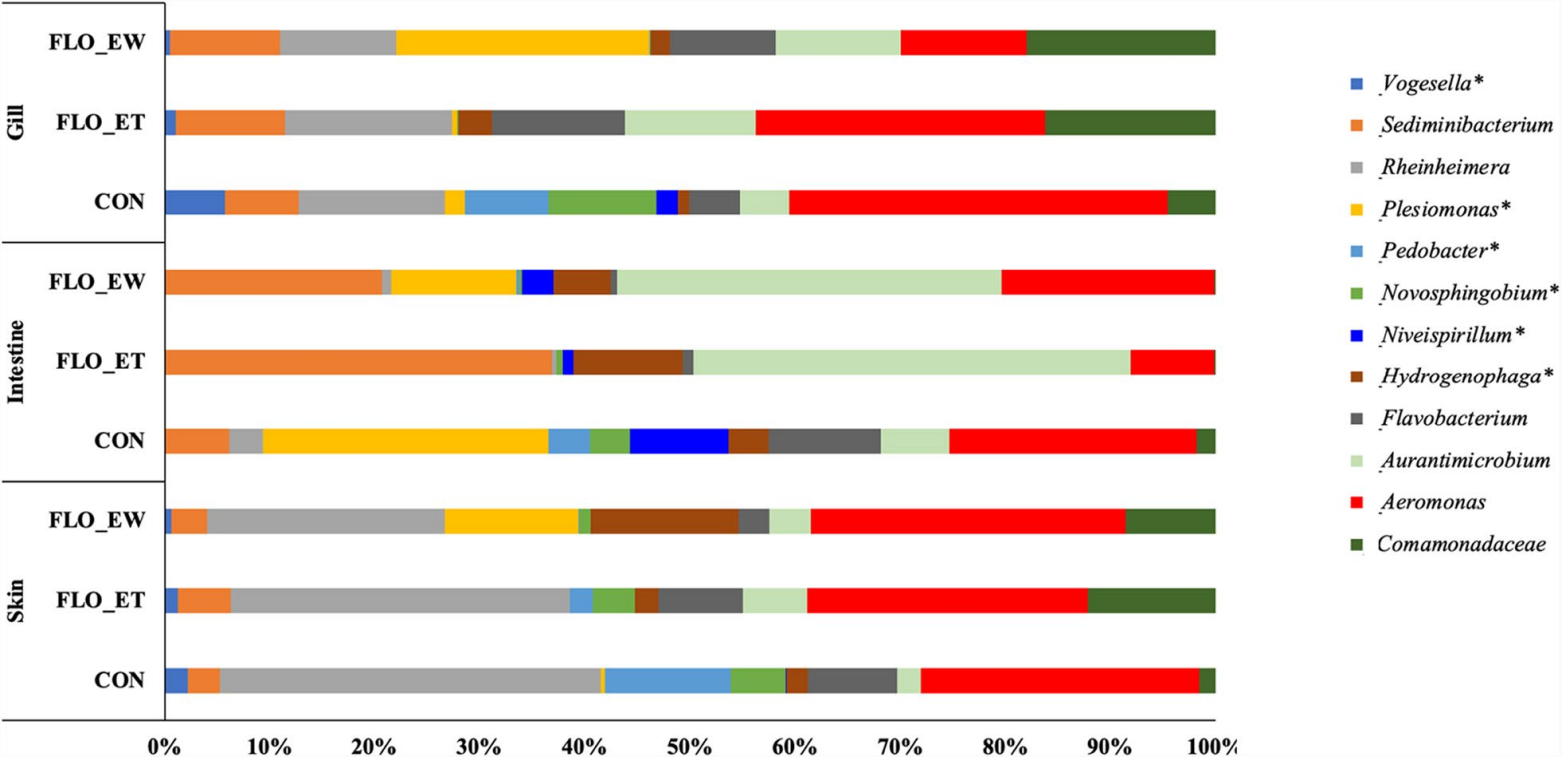
Similarities percentage (SIMPER) analysis screening top taxa (genera/family) driving variation in community composition associated with catfish gill, intestine, and skin due to the florfenicol treatment. Microbial members with an asterisk (*) indicate that the discriminatory genera significantly (*P*_adjust < 0.05) contributed to the differences in catfish microbial communities due to the florfenicol treatment (contribution to dissimilarity percentage ranged from 1.0 to 6.5%). Microbial members without an asterisk (*) indicate the important microbial members contributing to the dissimilarity among treatment groups (contribution to dissimilarity percentage ranged from 5.1 to 16.9%). CON: control group; FLO-ET: florfenicol-end of treatment; FLO-EW: florfenicol-end of withdrawal

Results from Similarities percentage (SIMPER) showed that *Aeromonas*, *Aurantimicrobium*, *Rheinheimera*, *Sediminibacterium*, *Plesiomonas*, *Comamonadaceae*, and *Flavobacterium* were classified as important contributing microbial members to the differences in catfish microbial communities caused by the florfenicol treatment (Bray–Curtis dissimilarity, contribution to dissimilarity percentage ranged from 5.1 to 16.9%). The other microbial members, denoting a significant contributing group, included *Novosphingobium*, *Plesiomonas*, *Pedobacter*, *Hydrogenophaga*, *Niveispirillum*, *Flavobacterium*, and *Vogesella*. These genera significantly (*P*_adjust < 0.05) contributed to the differences in catfish microbial communities caused by the florfenicol treatment (contribution to dissimilarity percentage ranged from 1.0% to 6.5%) (Fig. 5). Members that had their relative abundance significantly decreased due to the florfenicol treatment included *Vogesella*, *Plesiomonas*, *Pedobacter*, *Novosphingobium*, *Niveispirillum* and *Aeromonas* in catfish gill, *Rheinheimera*, *Plesiomonas*, *Pedobacter*, *Novosphingobium*, *Niveispirillum*, *Flavobacterium*, *Aeromonas*, and *Comamonadaceae* in catfish intestine, and *Vogesella, Rheinheimera, Plesiomonas*, *Pedobacter*, *Novosphingobium*, *Niveispirillum*, *Hydrogenophaga*, *Flavobacterium*, and *Aeromonas* in catfish skin.

### Profiling of ARGs in catfish intestine

**Fig. 6:**
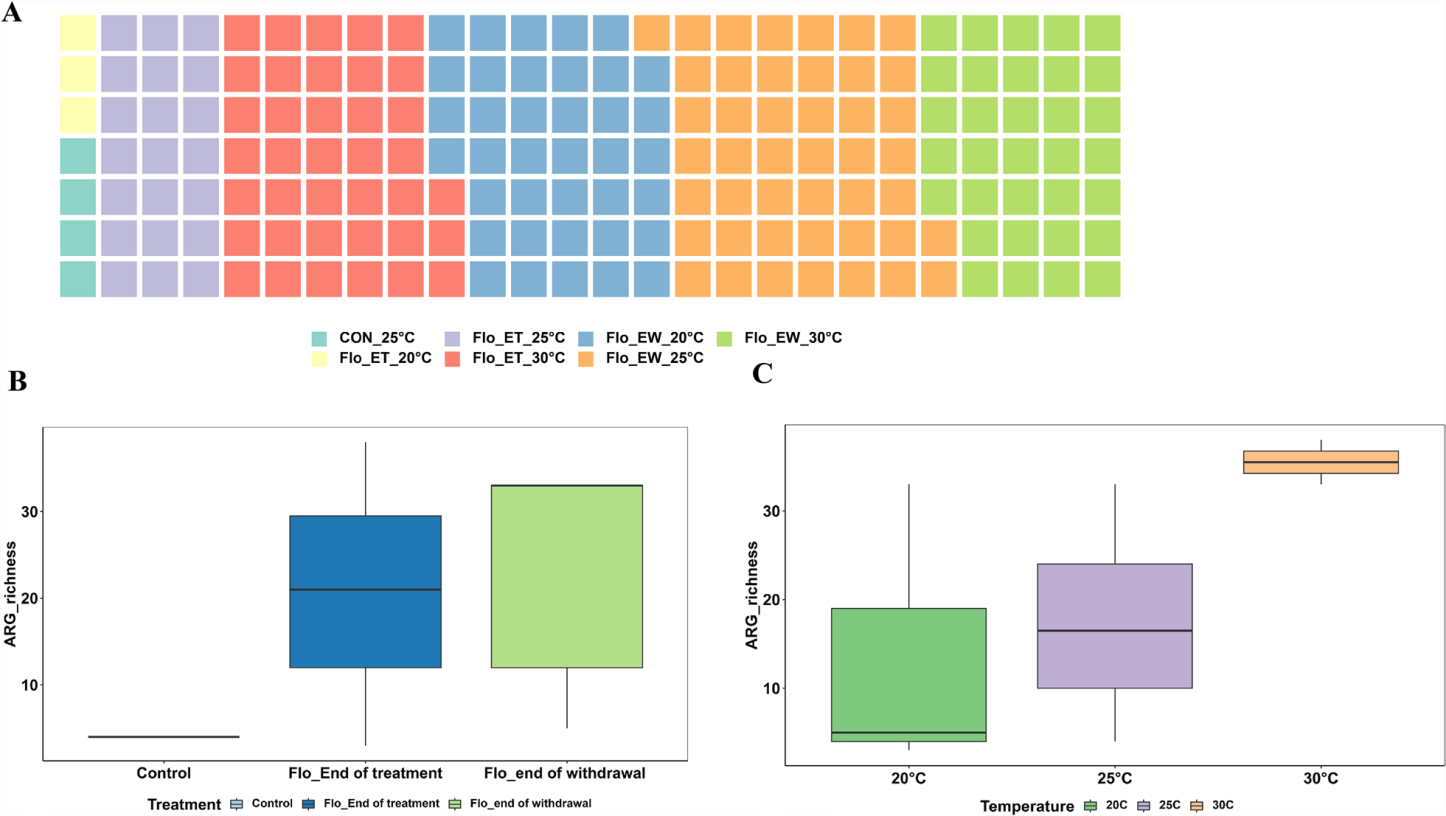
The resistome profile associated with catfish intestine samples with or without florfenicol treatment at 20, 25, and 30 °C. The types of antibiotic resistance gene (ARG) observed in different treatment groups based on the number of color blocks (A). Barplot of the number of unique ARGs (richness) observed in catfish intestine samples at different sampling times (B) and at different temperatures (C). CON: control; FLO_ET: florfenicol-end of treatment; FLO_EW: florfenicol-end of withdrawal

The goal of the metagenomic analysis was to take a closer look at the antibiotic profiles (ARGs) in the intestine samples and their association with the florfenicol treatment. During metagenomic analysis, a large portion of reads were aligned to the catfish host genome, and these host sequence reads were removed for down-stream ARG analysis. After host read removal, the remaining sequencing depth was insufficient to reliably assess abundance changes for ARGs within microbial community across samples. As such, only ARG types and richness were generated from the metagenomic part. Figure 6A shows the number of ARGs identified from different treatment combinations together with the controls. Each color square represents the presence of one ARG. The missing of squares for control groups at 20 and 30 °C indicates that ARG was undetected from the intestine samples collected from these two groups (Fig. 6A). A total of 111 ARGs belonging to 26 ARG groups, representing 17 antibiotic resistance mechanisms, were found in catfish intestine samples. The number of ARG types (ARG richness) present in catfish intestine samples increased significantly due to florfenicol treatment, with no significant difference observed between samples taken at the end of treatment and withdrawal (Fig. 6B). ARG richness increased with increasing rearing water temperatures (Fig. 6C).

## Discussion

In the present study, high-throughput analyses were used to evaluate the effects of florfenicol-medicated feed and water temperatures on the composition and ARG profiles associated with catfish gills, intestines, and skin. The effects were assessed both at the end of treatment and the end of withdrawal time.

A significant increase in intestinal microbial diversity was observed at the end of withdrawal period. The highest alpha diversity (Shannon index) was observed in catfish intestines. Except for the reduction of drug residue, this withdrawal period also allowed for the restoration of the normal microbiota. This increase indicated the restoration of the normal microbiota in catfish intestine. In accordance with our observation, an increase in microbial diversity was found in Atlantic salmon (*Salmo salar*) intestines after florfenicol treatment (at the end of treatment) [28]. In contrast, decreases in microbial diversity in Atlantic salmon or channel catfish due to oxytetracycline treatment or florfenicol treatment (no withdrawal data was reported) were reported in two other studies [16, 29]. This difference may be due to the difference in the used medicated feed, fish species, and other treatment conditions.

Florfenicol treatment and temperature had tissue-specific effects on the dynamics of catfish microbiota. In the gill, florfenicol significantly altered microbial structure only at 25 °C, suggesting a temperature-dependent sensitivity, while temperature alone affected the gill microbiota in untreated fish. In the intestine, florfenicol significantly altered microbial structure at 20 and 30 °C, with temperature effects observed during the withdrawal period, indicating the recovery of gut microbiota occurred with a delayed response. The skin microbiota was impacted by both florfenicol and temperature, with significant shifts observed at 20 and 30 °C, and temperature effects present before and during treatment but reduced after withdrawal. The differentiation of microbiota associated with different organs and types of tissue has been reported [30]. The phenotypic responses to environmental changes vary across the whole organism [30]. Similar to other multicellular eukaryotes, the interactions between catfish microbiota and the host can influence host fitness, particularly under environmental shifts; within a narrow temperature range, these interactions facilitate adaptation by altering microbial compositions across various catfish tissues [31, 32].

The predominant bacterial phyla shared among catfish gill, intestine, and skin were Proteobacteria (62%), Bacteroidetes (18%), Actinobacteriota (12%), Firmicutes (3%), Patescibacteria (2%), and Verrucomicrobiota (1%). Consistently, Proteobacteria, Bacteroidetes, and Firmicutes have been identified as the most abundant phyla in channel catfish gut microbiome [15, 16, 33, 34]. Although previous studies have focused primarily on the fish intestine microbiome, the predominance of Proteobacteria in catfish gill and skin is consistent with published research on other fish species, including grass carp, carnivorous southern catfish, seabass, seabream, and Atlantic salmon [35–37]. Studies have shown that Proteobacteria in salmon skin was associated with healthy salmonids [35]. In fish gill, Proteobacteria can serve as symbionts that play an important role in ammonia metabolism. Proteobacteria can help maintain nitrogen balance in fish by oxidizing ammonia to nitrite and nitrate [38, 39].

In the present study, Firmicutes was detected in catfish prior to the florfenicol treatment and became undetectable at the end of treatment in most treatment conditions. This change in Firmicutes suggested the potential inhibition effects of florfenicol on this group of bacteria. With the decrease of florfenicol during the withdrawal period, Firmicutes was dominant at the end of the withdrawal period at a higher tested temperature (30 °C). Firmicutes represent a large and diverse phylum; certain members, such as *Lactobacillus*, contribute beneficial metabolic activities, including the fermentation of indigestible carbohydrates [40–42]. One of the possible explanations for the observed increase in Firmicutes under higher temperature conditions indicates the recovery potential of the normal intestinal microbiota. However, overrepresentation of Firmicutes has also been linked to altered metabolic functions, energy balance, and potential inflammatory responses [43]. Other studies have reported similar results. Abdelhamed et al. [15] and Wang et al. [16] reported that Firmicutes present in the gut of channel catfish decreased after florfenicol treatments. The decrease in Firmicutes was linked to a reduction in *Lactobacillaceae*, *Streptococcaceae*, *Clostridiaceae1*, and *Bacillaceae* families in catfish [15].

At the genus level, florfenicol treatment decreased the relative abundance of *Plesiomonas*, *Pedobacter*, *Novosphingobium*, and *Aeromonas* in gills, intestines, and skin of catfish. Consistent with our findings, *Plesiomonas, Aeromonas, Lactococcus, Clostridium sensustricto, Romboutsia, Klebsiella, Turicibacter*, and *Lactobacillus* were decreased in florfenicol-fed channel catfish in comparison with control catfish [15]. Both *Aeromonas* and *Plesiomonas* have previously been regarded as opportunistic pathogens of catfish and other fish species [15, 44–46]. Species including *P. shigelloides*, *A. veronii*, and *A. hydrophila* have zoonotic importance and have been isolated from channel catfish and rainbow trout as fish pathogens [46–48]. *Pedobacter* is positively related to the ARGs (*Bla*_*TEM*_ and *intI-1*) in mariculture systems containing hybrid grouper and shrimps [35]. Some members of *Novosphingobium*, well-known for its diverse metabolic capabilities, can convert nitrate to nitrite. Despite not being reported in fish, *Novosphingobium* was widely isolated from freshwater, sediment, and fish culture ponds, demonstrating its excellent environmental adaptation [49, 50]. Applying florfenicol reduced potential pathogens; at the end of withdrawal, the discriminatory genera did not recover to the level seen in the catfish from the control group. This confirms that the florfenicol treatment effect can last until withdrawal, with an effect on catfish gut microbiota.

Given the prolonged impact of florfenicol on the fish intestinal microbiome, its effect on ARG was further evaluated for the intestine samples. Results showed that the richness of ARGs in catfish intestines increased at the end of the florfenicol treatment. Similarly, Sáenz et al. [51] found that ARGs were enriched at least 4.5 times in the intestine of the fish *Piaractus mesopotamicus* after florfenicol exposure [51]. The enrichment of ARGs may reflect microbial dysbiosis and altered susceptibility to opportunistic infections [52, 53]. The richness of ARGs in the catfish intestines did not decrease at the end of withdrawal, which is consistent with the microbial composition analysis. More research is needed to investigate the link between withdrawal duration and ARG decline. Other research has found that habitat and environmental factors can influence the ARG profile [9, 54]. Higher AMR levels of aquaculture-related bacteria were associated with warmer temperatures [9]. *Citrobacter*, an ARG host, was positively related to water temperature [54]. Finally, some opportunistic pathogens, such as *Streptococcus*, *Aeromonas*, and *Acinetobacter*, could be ARG hosts [54]. To reduce the spread of ARGs in aquaculture, it is critical to monitor the development of ARGs and opportunistic pathogens.

### Limitations

In this study, a large proportion of sequence reads from catfish host-genome were removed during host-decontamination steps, resulting in insufficient microbial sequencing depth or controls to assess ARG abundance changes within the microbial communities across treatment groups. In addition, statistical analysis between groups was not performed due to the limited sample size. However, the ARG-related analysis still provides detailed insight into the presence and types of resistance genes in catfish intestinal microbiota under florfenicol treatments. To improve future analyses, optimization of DNA isolation methods is necessary, particularly for fingerlings where gut content is limited and difficult to separate from host tissues. Selective enrichment for microbial DNA prior to sequencing, along with generating higher sequencing depth, would also help reduce host DNA interference and enable more robust assessment of ARG dynamics.

## Conclusions

In summary, florfenicol treatment can alter the catfish microbiota, with effects persisting through the withdrawal period. Although the withdrawal period effectively reduced drug residues, restoration of normal microbiota was only partial. Microbial disruption remained evident, and the enrichment of ARG was detected during the withdrawal period. This observation indicated a potential risk of resistance spreading, possibly through horizontal gene transfer within aquaculture systems. Future studies are needed to conduct more in-depth analyses of ARG dynamics and evaluate how to better mitigate the risk of AMR dissemination in aquaculture environments.

## Supplementary Information

Additional file 1.

Additional file 2.

Additional file 3.

## Data Availability

NCBI Sequence Read Archive (SRA) within Bioproject #PRJNA1240677.
