# Supplementary material for: The impact of florfenicol treatment on the microbial populations present in the gill, intestine, and skin of channel catfish (Ictalurus punctatus)
Source: Anim Microbiome. 2025 Jun 20;7:68. doi: 10.1186/s42523-025-00433-9 (PMC12180268; doi:10.1186/s42523-025-00433-9)
Supplement: Supplementary file 1 — Additional file 1. [file 42523_2025_433_MOESM1_ESM.pptx]

## Slide 1
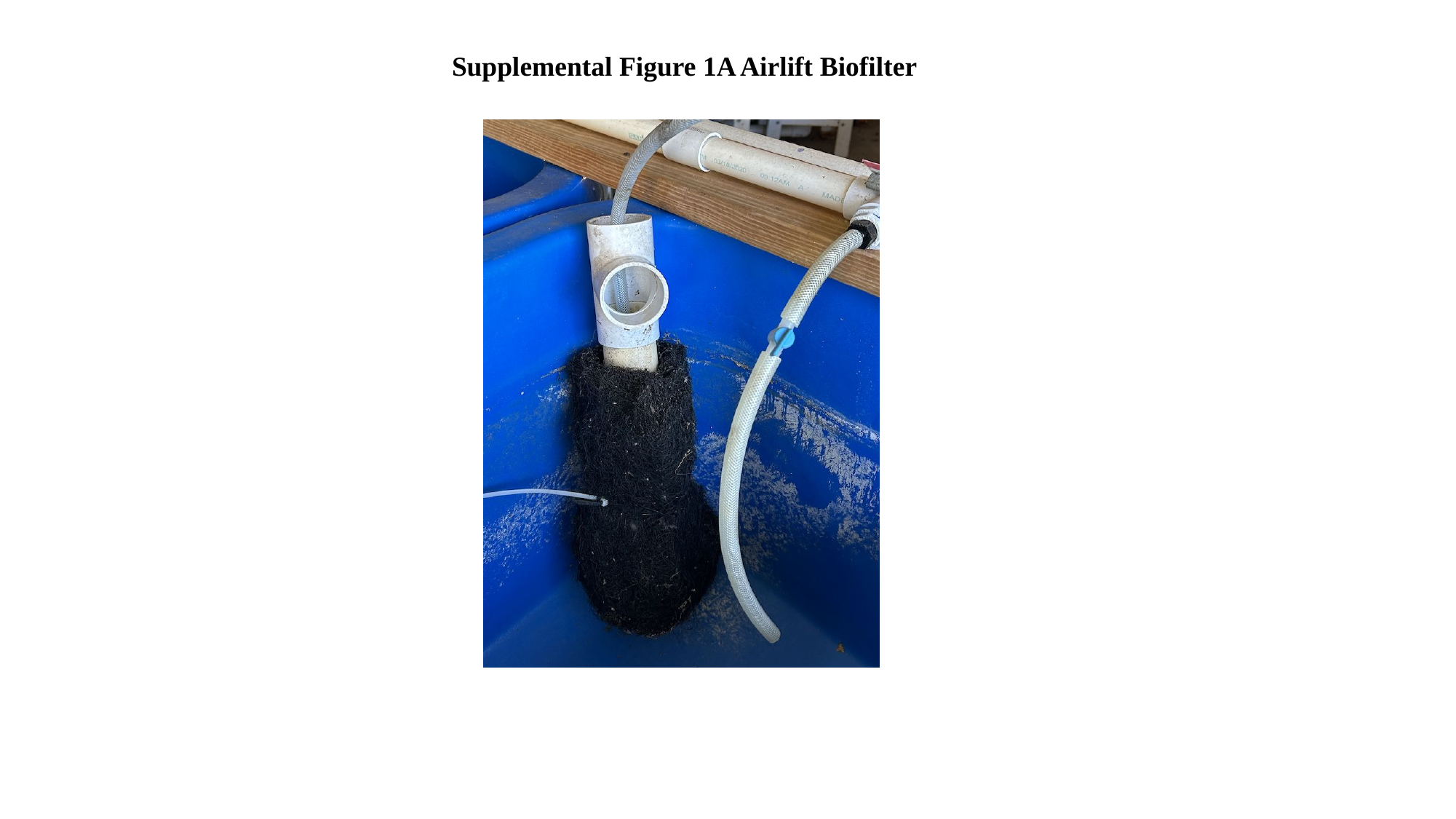

Supplemental Figure 1A Airlift Biofilter

## Slide 2
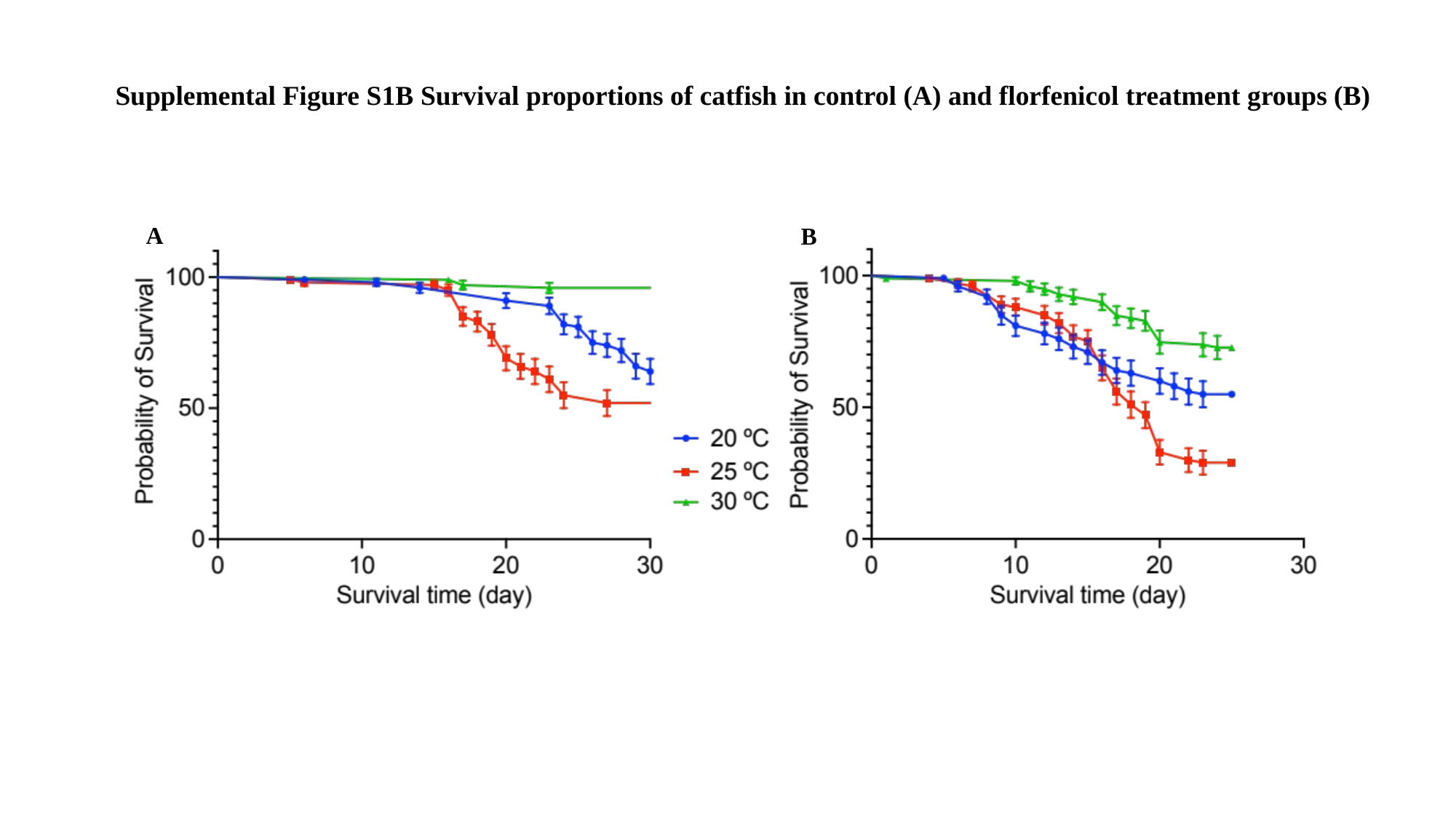

Supplemental Figure S1B Survival proportions of catfish in control (A) and florfenicol treatment groups (B)
A
B
