# Supplementary material for: The impact of florfenicol treatment on the microbial populations present in the gill, intestine, and skin of channel catfish (Ictalurus punctatus)
Source: Anim Microbiome. 2025 Jun 20;7:68. doi: 10.1186/s42523-025-00433-9 (PMC12180268; doi:10.1186/s42523-025-00433-9)
Supplement: Supplementary file 2 — Additional file 2. [file 42523_2025_433_MOESM2_ESM.pptx]

## Slide 1
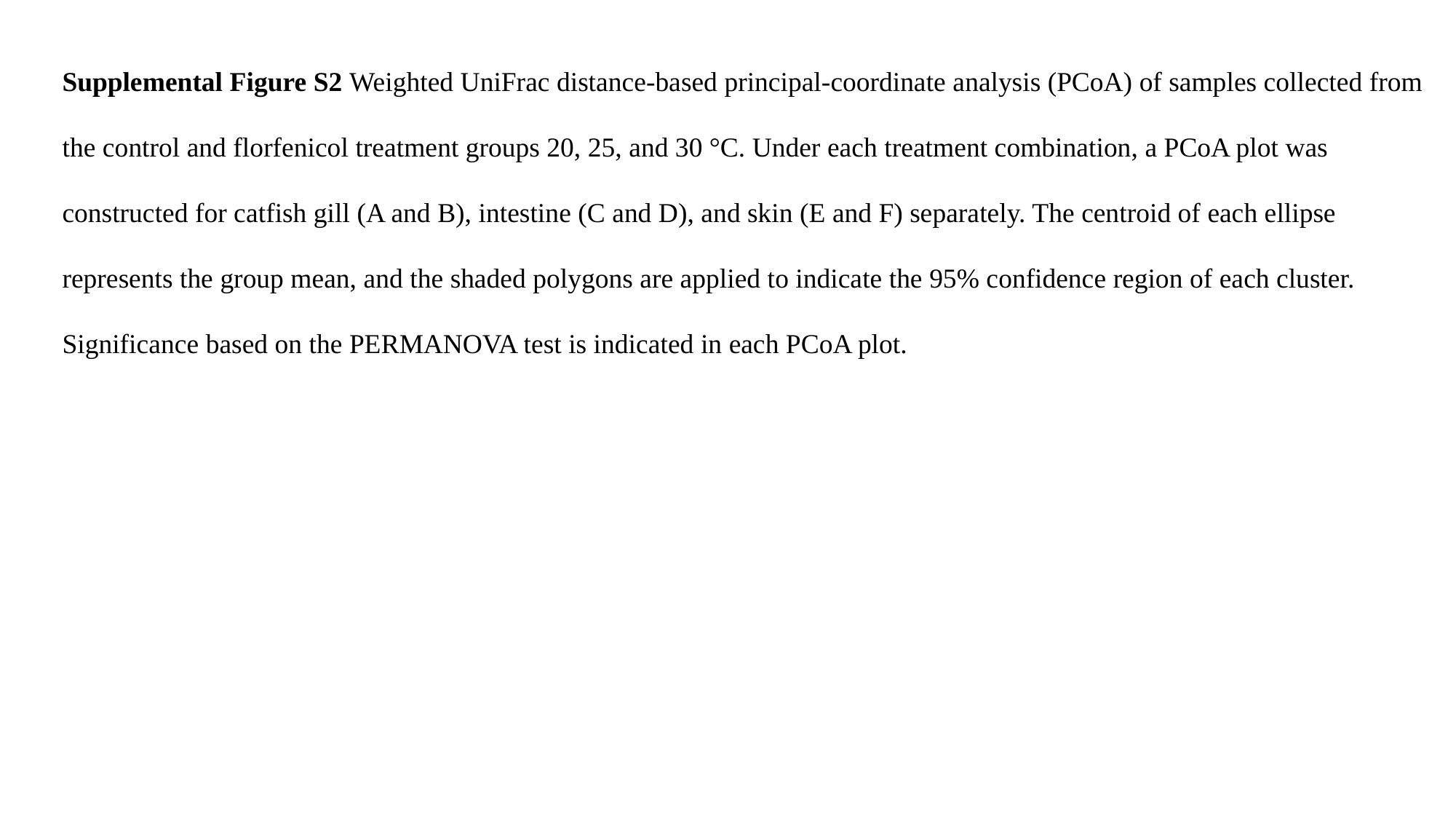

Supplemental Figure S2 Weighted UniFrac distance-based principal-coordinate analysis (PCoA) of samples collected from the control and florfenicol treatment groups 20, 25, and 30 °C. Under each treatment combination, a PCoA plot was constructed for catfish gill (A and B), intestine (C and D), and skin (E and F) separately. The centroid of each ellipse represents the group mean, and the shaded polygons are applied to indicate the 95% confidence region of each cluster. Significance based on the PERMANOVA test is indicated in each PCoA plot.

## Slide 2
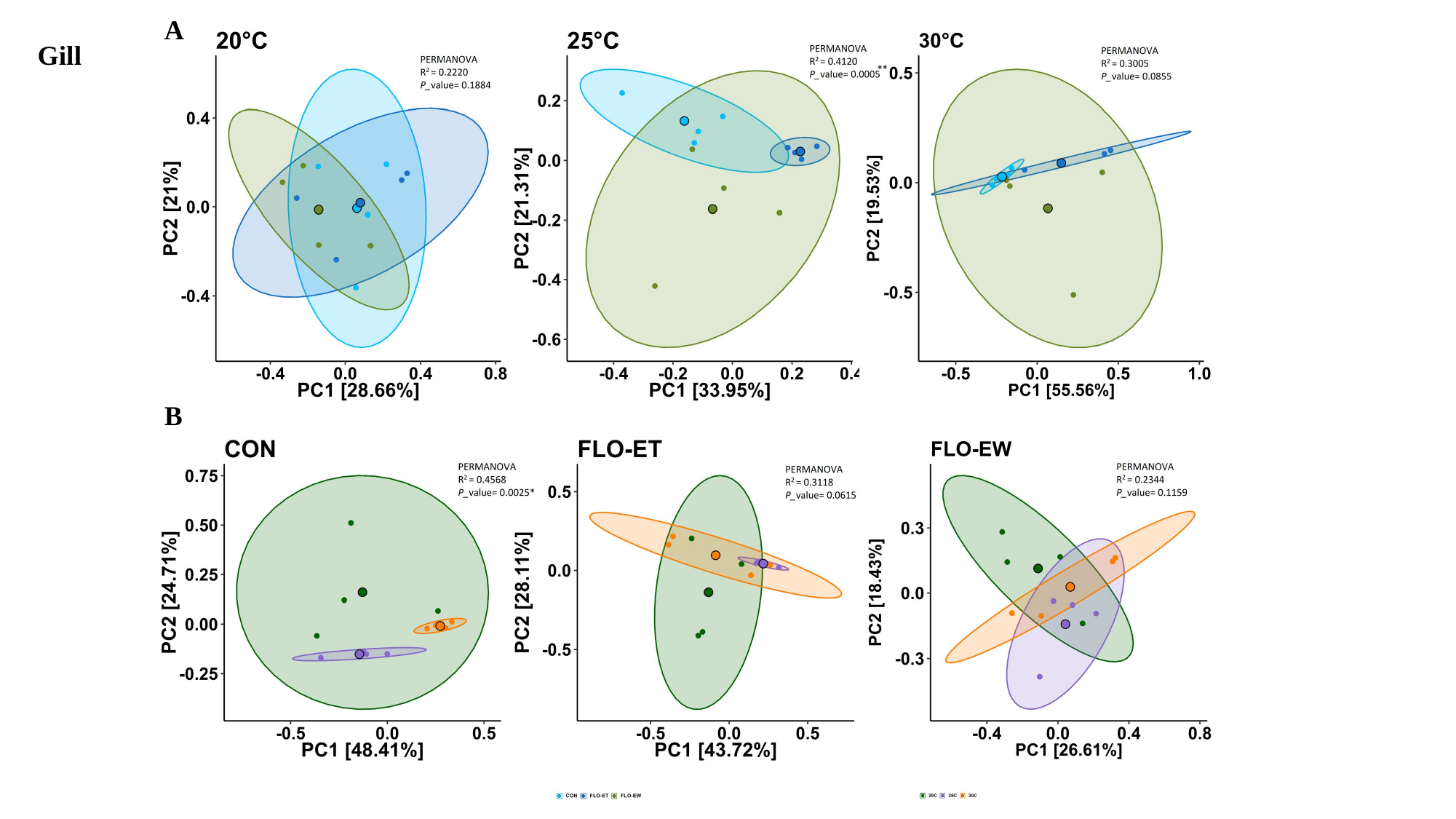

A
Gill
B

## Slide 3
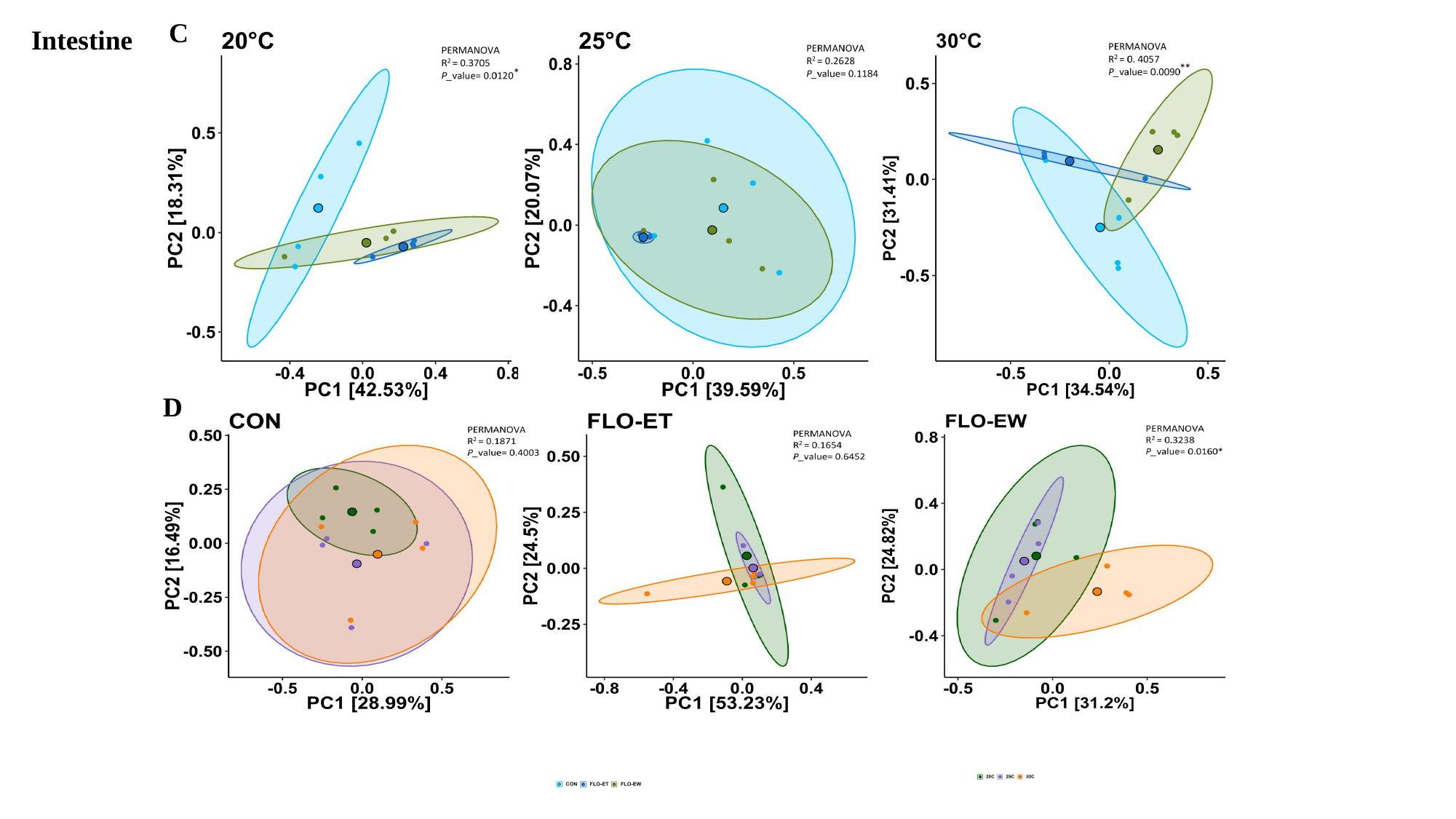

C
Intestine
D

## Slide 4
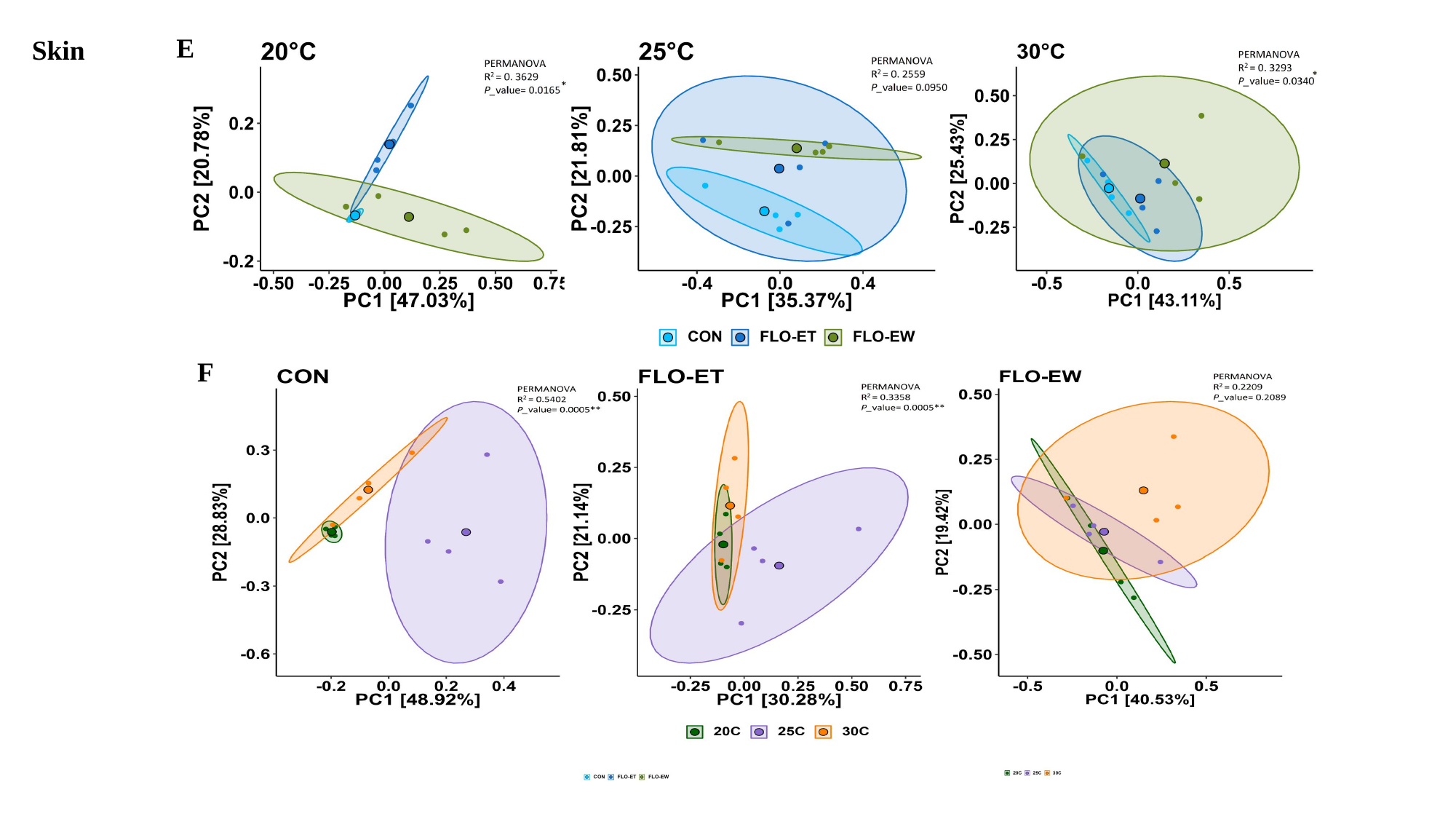

E
Skin
F
