# Supplementary material for: The impact of florfenicol treatment on the microbial populations present in the gill, intestine, and skin of channel catfish (Ictalurus punctatus)
Source: Anim Microbiome. 2025 Jun 20;7:68. doi: 10.1186/s42523-025-00433-9 (PMC12180268; doi:10.1186/s42523-025-00433-9)
Supplement: Supplementary file 3 — Additional file 3. [file 42523_2025_433_MOESM3_ESM.docx]

**Supplemental Table S1 Water pH monitoring during the experimental period**

| Sample ID | 10/25/2020 | 10/30/20 | 11/4/20 | 11/10/20 | 11/18/20 | 11/25/24 | 12/2/20 | 12/9/20 |
| --- | --- | --- | --- | --- | --- | --- | --- | --- |
| Control-20-T1 | 9.18 | 8.79 | 8.87 | 8.99 | 8.60 | 8.87 | 8.96 | 9.14 |
| Control-20-T2 | 9.17 | 9.10 | 8.91 | 9.08 | 8.62 | 8.85 | 8.95 | 9.09 |
| Control-20-T3 | 9.08 | 9.05 | 8.97 | 8.94 | 8.63 | 8.81 | 8.93 | 9.11 |
| Control-20-T4 | 9.05 | 8.99 | 9.05 | 8.96 | 8.63 | 8.92 | 8.92 | 9.12 |
| Control-25-T1 | 9.00 | 8.92 | 9.02 | 8.93 | 8.64 | 8.66 | 8.98 | 9.13 |
| Control-25-T2 | 8.97 | 8.90 | 8.99 | 8.85 | 8.61 | 8.65 | 8.88 | Not tested |
| Control-25-T3 | 8.96 | 8.90 | 8.99 | 8.87 | 8.65 | 8.73 | 8.92 | 9.13 |
| Control-25-T4 | 8.94 | 8.91 | 8.98 | 8.83 | 8.66 | 8.74 | 8.88 | 9.16 |
| Control-30-T1 | 8.93 | 8.89 | 8.96 | 8.91 | 8.64 | 8.70 | 8.90 | 9.16 |
| Control-30-T2 | 8.90 | 8.89 | 8.91 | 8.93 | 8.66 | 8.81 | 8.97 | 9.16 |
| Control-30-T3 | 8.88 | 8.89 | 8.93 | 8.96 | 8.70 | 8.78 | 8.88 | 9.14 |
| Control-30-T4 | 8.89 | 8.88 | 8.95 | 8.97 | 8.75 | 8.81 | 8.90 | 9.15 |
| Florfenicol-20-T1 | 8.93 | 8.92 | 8.88 | 9.07 | 8.71 | 9.16 | 8.94 | 9.16 |
| Florfenicol-20-T2 | 8.92 | 8.90 | 8.87 | 9.05 | 8.74 | 9.15 | 8.96 | 9.14 |
| Florfenicol-20-T3 | 8.90 | 8.90 | 8.85 | 9.00 | 8.73 | 9.15 | 9.07 | 9.13 |
| Florfenicol-20-T4 | 8.91 | 8.89 | 8.84 | 8.99 | 8.72 | 9.13 | 8.95 | 9.07 |
| Florfenicol-25-T1 | 8.89 | 8.85 | 8.85 | 9.11 | 8.69 | 9.11 | 8.88 | 9.11 |
| Florfenicol-25-T2 | 8.89 | 8.86 | 8.86 | 9.08 | 8.69 | 9.06 | 8.86 | 9.08 |
| Florfenicol-25-T3 | 8.87 | 8.89 | 8.89 | 9.04 | 8.73 | 9.06 | 8.86 | 9.01 |
| Florfenicol-25-T4 | 8.87 | 8.88 | 8.86 | 8.95 | 8.70 | 9.00 | 8.92 | 9.09 |
| Florfenicol-30-T1 | 8.86 | 8.86 | 8.86 | 8.94 | 8.70 | 8.98 | 9.08 | 9.04 |
| Florfenicol-30-T2 | 8.84 | 8.86 | 8.89 | 8.89 | 8.67 | 8.96 | 9.09 | 9.00 |
| Florfenicol-30-T3 | 8.86 | 8.88 | 8.89 | 8.89 | 8.71 | 9.02 | 9.06 | 9.02 |
| Florfenicol-30-T4 | 8.84 | 8.88 | 8.88 | 8.88 | 8.70 | 9.02 | 9.03 | 9.04 |

**Supplemental Table S2 Unionized ammonia (mg/L) monitoring during the experimental period**

| Sample ID | 10/25/2020 | 10/30/20 | 11/4/20 | 11/10/20 | 11/18/20 | 11/25/24 | 12/2/20 | 12/9/20 |
| --- | --- | --- | --- | --- | --- | --- | --- | --- |
| Control-20-T1 | 0.77 | 0.20 | 0.20 | 0.07 | 0.03 | 0.05 | 0.07 | 0.10 |
| Control-20-T2 | 0.77 | 0.39 | 0.28 | 0.07 | 0.07 | 0.05 | 0.07 | 0.07 |
| Control-20-T3 | 0.57 | 0.28 | 0.14 | 0.07 | 0.03 | 0.05 | 0.07 | 0.10 |
| Control-20-T4 | 0.57 | 0.28 | 0.28 | 0.07 | 0.07 | 0.07 | 0.07 | 0.10 |
| Control-25-T1 | 0.72 | 0.36 | 0.36 | 0.09 | 0.05 | 0.05 | 0.09 | 0.12 |
| Control-25-T2 | 0.72 | 0.36 | 0.36 | 0.07 | 0.05 | 0.05 | 0.07 | Not tested |
| Control-25-T3 | 0.72 | 0.36 | 0.36 | 0.07 | 0.05 | 0.07 | 0.09 | 0.12 |
| Control-25-T4 | 0.72 | 0.36 | 0.36 | 0.07 | 0.05 | 0.07 | 0.07 | 0.12 |
| Control-30-T1 | 0.89 | 0.34 | 0.22 | 0.11 | 0.06 | 0.08 | 0.11 | 0.14 |
| Control-30-T2 | 0.89 | 0.34 | 0.45 | 0.11 | 0.06 | 0.08 | 0.11 | 0.14 |
| Control-30-T3 | 0.67 | 0.34 | 0.22 | 0.11 | 0.08 | 0.08 | 0.08 | 0.14 |
| Control-30-T4 | 0.67 | 0.34 | 0.45 | 0.11 | 0.08 | 0.08 | 0.11 | 0.14 |
| Florfenicol-20-T1 | 0.57 | 0.28 | 0.20 | 0.07 | 0.05 | 0.10 | 0.07 | 0.10 |
| Florfenicol-20-T2 | 0.57 | 0.28 | 0.20 | 0.07 | 0.05 | 0.10 | 0.07 | 0.10 |
| Florfenicol-20-T3 | 0.57 | 0.28 | 0.10 | 0.07 | 0.05 | 0.10 | 0.07 | 0.10 |
| Florfenicol-20-T4 | 0.57 | 0.20 | 0.20 | 0.07 | 0.05 | 0.10 | 0.07 | 0.07 |
| Florfenicol-25-T1 | 0.53 | 0.26 | 0.13 | 0.12 | 0.05 | 0.12 | 0.07 | 0.12 |
| Florfenicol-25-T2 | 0.53 | 0.26 | 0.07 | 0.09 | 0.05 | 0.09 | 0.07 | 0.09 |
| Florfenicol-25-T3 | 0.53 | 0.26 | 0.07 | 0.09 | 0.07 | 0.09 | 0.07 | 0.09 |
| Florfenicol-25-T4 | 0.53 | 0.26 | 0.00 | 0.09 | 0.09 | 0.09 | 0.09 | 0.09 |
| Florfenicol-30-T1 | 0.67 | 0.20 | 0.34 | 0.11 | 0.06 | 0.11 | 0.11 | 0.11 |
| Florfenicol-30-T2 | 0.67 | 0.20 | 0.00 | 0.08 | 0.06 | 0.11 | 0.11 | 0.11 |
| Florfenicol-30-T3 | 0.67 | 0.20 | 0.00 | 0.08 | 0.08 | 0.11 | 0.11 | 0.11 |
| Florfenicol-30-T4 | 0.67 | 0.20 | 0.08 | 0.08 | 0.06 | 0.11 | 0.11 | 0.11 |

**Supplemental Table S3 Nitrite (mg/L) monitoring during the experimental period**

| Sample ID | 10/25/2020 | 10/30/20 | 11/4/20 | 11/10/20 | 11/18/20 | 11/25/24 | 12/2/20 | 12/9/20 |
| --- | --- | --- | --- | --- | --- | --- | --- | --- |
| Control-20-T1 | 0 | 0 | 0 | 5 | 0.25 | 0.25 | 2 | 0.25 |
| Control-20-T2 | 0 | 0 | 0.5 | 5 | 0.25 | 0 | 2 | 0 |
| Control-20-T3 | 0 | 0 | 0.25 | 0.5 | 0.25 | 1 | 2 | 0 |
| Control-20-T4 | 0 | 0.25 | 0.25 | 1 | 0.25 | 2 | 0.5 | 0.25 |
| Control-25-T1 | 0 | 0 | 0.5 | 1 | 0.25 | 0.5 | 0.5 | 0 |
| Control-25-T2 | 0 | 0 | 0.25 | 5 | 0.25 | 0.25 | 0.25 | Not tested |
| Control-25-T3 | 0 | 0.25 | 0.25 | 0.25 | 0.5 | 0 | 0.25 | 0 |
| Control-25-T4 | 0 | 0 | 0.5 | 1 | 0.25 | 0.5 | 0 | 0 |
| Control-30-T1 | 0 | 0.25 | 1 | 0.5 | 0.25 | 0 | 0 | 0 |
| Control-30-T2 | 0 | 0.25 | 0.25 | 5 | 0.25 | 2 | 0 | 0 |
| Control-30-T3 | 0 | 0.25 | 1 | 2 | 0.25 | 1 | 0.25 | 0 |
| Control-30-T4 | 0 | 0 | 0 | 5 | 0.25 | 0.25 | 0 | 0 |
| Florfenicol-20-T1 | 0 | 0 | 0.5 | 1 | 0.5 | 0.5 | 0.25 | 0.25 |
| Florfenicol-20-T2 | 0 | 0 | 1 | 0.5 | 0.5 | 0.25 | 0.25 | 0 |
| Florfenicol-20-T3 | 0 | 0 | 1 | 0.5 | 0 | 1 | 0.25 | 0 |
| Florfenicol-20-T4 | 0 | 0 | 1 | 0.5 | 0.5 | 0.5 | 0 | 0 |
| Florfenicol-25-T1 | 0 | 0 | 0.5 | 0 | 0.25 | 0.25 | 0 | 0 |
| Florfenicol-25-T2 | 0 | 0.5 | 1 | 0 | 0.25 | 0 | 0.25 | 0 |
| Florfenicol-25-T3 | 0 | 0.25 | 0.5 | 0.5 | 0.5 | 0.25 | 0.25 | 0 |
| Florfenicol-25-T4 | 0 | 0.5 | 1 | 0.5 | 0.25 | 0.25 | 0.25 | 0 |
| Florfenicol-30-T1 | 0 | 0.25 | 0.25 | 2 | 0.25 | 0.5 | 0.5 | 0 |
| Florfenicol-30-T2 | 0 | 0.5 | 0.5 | 0.5 | 0.25 | 0 | 0 | 0 |
| Florfenicol-30-T3 | 0 | 0.5 | 0.5 | 0.5 | 0 | 0 | 0 | 0 |
| Florfenicol-30-T4 | 0 | 0.25 | 1 | 0 | 0.25 | 0.25 | 0 | 0 |

**Supplemental Table S4** Precursor ion, quantifier and qualifier product ions, fragmentor voltage, and collision energy (CE) for antibiotic (Florfenicol) standards.

| Antibiotics | Abbreviations | Precursor Ion | Fragmentor voltage | MRM 1 (CE) | MRM 2 (CE) | Polarity |
| --- | --- | --- | --- | --- | --- | --- |
| Florfenicol | FF | 355.9 | 125 | 185.1 (10) | 118.7 (30) | Negative |

**Supplemental Table S5 Sample information**

| Sampling time | Treatment | Temperature | 16S rRNA gene  (Gill, Intestine, and Skin) | Metagenomics  (Intestine only) |
| --- | --- | --- | --- | --- |
| Day 0 | Control tanks | 20 °C | 12 | 2 |
|  |  | 25 °C | 12 | 2 |
|  |  | 30 °C | 12 | 2 |
| Day 12 | Florfenicol tanks | 20 °C | 12 | 2 |
|  |  | 25 °C | 12 | 2 |
|  |  | 30 °C | 12 | 2 |
| Day 24 | Florfenicol tanks | 20 °C | 12 | 2 |
|  |  | 25 °C | 12 | 2 |
|  |  | 30 °C | 12 | 2 |
| Total |  |  | 108 | 18 |

**Supplemental Table S6** Catfish weight measured at different sampling points. *n* = 20 for each sampling time at every treatment temperature.

|  |  | Fish Weight (g) | |
| --- | --- | --- | --- |
| Treatment/Sampling time | Temperature | Median | Mean ± SD |
| Control/Day 0 | 20 °C | 5.7 | 6.5 ± 3.1 |
|  | 25 °C | 7.3 | 7.8 ± 2.4 |
|  | 30 °C | 6.9 | 7.8 ± 5.0 |
| End of treatment/Day 12 | 20 °C | 5.6 | 5.9 ± 1.7 |
|  | 25 °C | 5.2 | 5.5 ± 2.1 |
|  | 30 °C | 7.9 | 8.7 ± 2.3 |
| End of withdrawal/Day 24 | 20 °C | 6.2 | 6.8 ± 2.5 |
|  | 25 °C | 4.7 | 4.9 ± 2.0 |
|  | 30 °C | 7.9 | 8.7 ± 2.3 |
